# The Effect of Reflexology on Pain Intensity and Duration of Labor on Primiparas

**Published:** 2011-07-01

**Authors:** M Dolatian1, A Hasanpour, Sh Montazeri, R Heshmat, H Alavi Majd

**Affiliations:** 1 Department of Midwifery, Shahid Beheshti University of Medical Sciences, Tehran, Iran; 2 Department of Medicine and Health, International Medical University (IMU), Bukit, Jalil, Kualalumpur, Malaysia; 3 Acupuncture and Reflexology Specialist, Tehran, Iran; 4 Department of Biostatistics, Shahid Beheshti University of Medical Sciences, Tehran, Iran

**Keywords:** Reflexology, Primiparas, Pain intensity, Duration of labor

## Abstract

**Background:**

Reflexology is an ancient, mild and non-invasive technique, used widely as one of the non-pharmacological methods for pain relief. The aim of this research was to determine the effect of reflexology on pain intensity as well as to determine the duration of labor in primiparas.

**Methods:**

In 2008, a randomized clinical trial study was conducted randomly enrolling 120 parturient women with low risk pregnancy into three groups in Shahid Akbarabadi Hospital, Tehran, Iran. The first group received 40 minutes of reflexology at the beginning of active phase (4-5 cm cervical dilatation). Emotional support was offered for the second group in the same stage of pregnancy and with the same duration. The third group received only routine care during labor. Pain severity was evaluated with visual analogue scale (0 to 10 cm). In all groups, pregnant women were asked to evaluate the severity of pain experienced before and after intervention and also at cervical dilatations of 6-7 cm and 8-10 cm respectively. Data were collected through the numerical pain scale.

**Results:**

Pain intensity at all the three stages of cervical dilatation was significantly lower in the reflexology group. During the 4-5 cm dilatation stage, women in the supported group reported less severe pain compared to those receiving routine care, but no significant differences at the later stages of labor. This indicates that reflexology could decrease the duration of first, second and third stages of labor.

**Conclusion:**

Our findings showed that reflexology can be useful to decrease the pain intensity as well as duration of labor.

## Introduction

Mankind has experienced pain since the beginning of time. According to the definition of the International Association of Pain, pain is an unpleasant and mentalemotional experience, which is accompanied with tissue damage[1]. One of the most severe pains known to man is labor pain[2], which is synonymous with parturition. During delivery, excessive pain leads to fear and anxiety. This stimulates the sympathetic nervous system to increase catecholamine secretion leading to increased blood levels of hormones such as epinephrine. These will further intensify the pain, and potentially prolong the first and second stages of labor, thus resulting in a very unpleasant experience of childbirth [3].Additionally, prolonged first stage of labor is associated with fetal complications, including head compression, impaired oxygen supply, low Apgar score and ultimately fetal death [4][5][6]. Cheng et al. reported that although duration of the second stage of labor is not associated with poor neonatal outcome, prolonged second stage of labor is accompanied by increasing serious complications such as perineal trauma and increased caesarean delivery after first hour of the second stage of labor [7].

Over the years, mankind had devised many methods to combat pain. Pain relief methods can be divided into two main groups: pharmacological and nonpharmacological ones. One of the most significant limitations associated with pharmacological pain relief is that almost every drug that is used for labor analgesia in the mother can pass through the placenta. This has deleterious effects on both the mother and the fetus. The fetus’ respiratory system may be weakened, and the mother experience long labor and reflex disorder in the second stage of delivery [8]. Three principles that are essential to relieve pain in midwifery include simplicity, safety and maintaining fetal homeostasis [9], and the non-pharmacological methods satisfy all of these. There is no effect on delivery, and no maternal or fetal side effects.

Reflexology is a noninvasive and nonpharmacological method of pain relief. It is based on a system of zones and reflex areas on the feet and palms that reflect an image of the entire body (including muscle, nerve, gland and bone) in exactly the same order and position as in the body[10]. Although there is still ambiguity regarding the mechanism of action of reflexology, some theories have been proposed. These include the gate control theory of pain (the pain gate theory), neural impulse theory, increased secretion of endorphins and encephalins which assist relieving of pain, improved lymphatic nerve and blood flow and consequently increased excretion of toxins from the body [10][11].

Reflexology has been used during pregnancy for treatment of various physiological problems such as nausea and vomiting, constipation, edema[12], fatigue,[13] headache and to help breastfeeding[14][15]. The lack of sufficient research on the effectiveness of reflexology in relieving labor pain, has hindered the implementation of nonpharmacological methods of pain in our country with an increasing growth of elective cesarean section and maternal desire without medical indi-cation, there is need for more research in alternative management of pain. We aimed to study the effect of foot reflexology on pain of labor in primiparous women, as a practical strategy for minimizing labor pain without the use of drugs.

## Materials and Methods

This study was a clinical trial with three study groups on primiparous women attending the maternity ward in Shahid Akbar Abadi Hospital, Tehran, Iran in 2008. The study protocol was approved by the Research and Ethics Committee of the Faculty of Nursing and Midwifery, Shahid Beheshti University of Medical Sciences. The purpose of the study was explained to all the participating women by researchers and all of the recruited women had given informed (signed) consent.

Subjects were recruited based on the inclusion and exclusion criteria which specified low risk women with no medical or obstetric complications. Altogether 120 singleton pregnant women aged 18-35 years old at the gestational period between 37-42 weeks satisfying this and other relevant criteria were enrolled. The random allocation software for clinical trial was then used to assign the subject into three groups namely reflexology, support and routine care. Sampling days was selected randomly for each group.

**Figure 1:**
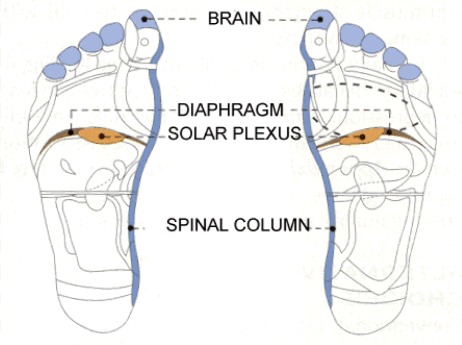
Reflexology session, feet areas

For the reflexology session, thumb and index fingers were used to work on the feet. First mild massage was given on all feet and then pressure was applied on concerned and specified regions. As Figure 1 shows, these areas were: 1) Pituitary gland, in the centre of the thumb; 2) Solar plexus, almost four fingers width below the base of the fingers of the feet, located in the center (middle of diaphragm); 3) Lumbar and sacral spine (spinal cord region) and 4) Genital area, below the ankle.

The work method was done based on the reference book of reflexology [16] and approved by a specialist in reflexology. When the subjects were at a dilation of 4-5 cm (active phase), reflexology was performed by the researcher once and for 20 minutes on each foot (total 40 minutes). Then the researcher followed subjects until after delivery. The support group received spiritual, emotional and verbal support from the researcher for 40 minutes beginning at the 4 cm dilation stage, into the active phase of the labor. The routine care group benefited from routine care only. Pain severity was evaluated with visual analogue scale (pain ruler 0 to 10 cm). Data was recorded on the information sheet provided. The pain scores before intervention and immediately after intervention for reflexology, and support groups; and for the same period for the routine care group were recorded. Pain scores were also recorded in cervical dilation of 6-7 cm and 8-10 cm. Duration of three stage of labor was explained as below: First stage: When the cervix was about 4 cm dilated and till 10 cm, second stage: From 10 dilatation to delivery of baby, third stage: Duration between delivery of baby and delivery of placenta.

Statistical analysis using SPSS (version 11.5, Chicago, IL, USA) was performed. The mean of quantitative variables in three study groups was compared by ANOVA, Post-hoc and Kruskal Wallis tests. A level of p<0.05 was considered statistically significant.

## Results

Sample characteristics of study subjects are shown in Table 1. The mean age of subjects in the study groups was 22.9±3.85 years. Pain intensity before and after intervention is shown inFigure 2. The mean pain score before intervention was calculated in three groups and Kruskal Wallis test showed no significant difference among the study groups. In cervical dilation 4-5 cm, the mean pain severity was 4.5±1.06 in reflexology group, 6.25±0.84 in the support group and 7.23±0.83 in the routine care group. The statistical tests showed significant difference at this stage among the study groups (p<0.001). The results of the Mann-Whitney test also showed a significant differences between reflexology group and support group, reflexology group and routine care group as well as support group and routine care group (p<0.001, p<0.001, p<0.001 respectively).

**Table 1:** Demographic characteristics of the 3 groups in terms of mean and standard deviation (SD)

**Characteristics**	**Reflexology**		**Support**		**Routine**		**Significance**
	**Mean**	**SD**	**Mean**	**SD**	**Mean**	**SD**	
Age	22.78	3.21	22.95	3.57	22.90	3.85	NS^a^
BMI(kg/m^2^)	23.79	1.83	23.32	1.80	23.48	1.97	NS
Gestational Age by sonography (weeks)	39	1.36	38	1.37	39	1.24	NS

^a^ Not significant

**Figure 2:**
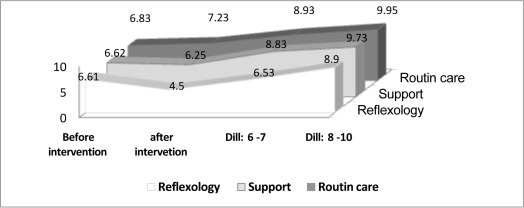
Pain intensity before and after intervention

During dilation of 6-7 cm as well as 8-10 cm, the intensity of pain was significantly lower in reflexology group compared to the other two groups (p<0.001). No statistical difference was observed between support group and routine care group in terms of pain intensity in dilation 6-7 cm as well as 8-10 cm (p=0.07).

The mean duration of three stages of labor in three study groups was calculated (Table 2).

**Table 2:** The mean duration of three stages of labor in three study groups.

**Groups**	**Reflexology**	**Support**	**Routine Care**	**P value**
**Variables**	**Mean±SD**	**Mean±SD**	**Mean±SD**	
Duration of first stage (Minutes)	166.88±42.02	207.13±64.41	229.13±51.52	0.001
duration of second stage (Minutes)	25.18±17.24	47.63±2.087	55.63±27.29	0.001
duration of third stage (Minutes)	3.33±1.34	7.55±6.29	7.93±12.15	0.001

The mean duration of three stages of labor was compared using ANOVA and the results indicated significant difference among the study groups (p<0.001). After doing Post-hoc test, the results demonstrated that the length of the first stage of labor was significantly lower in reflexology group compared to support group as well as routine care group (p<0.003 and p<0.001 respectively). But no statistical difference was seen between support group and the routine care group. It was elicited that the duration of second and third stages of labor were also significantly lower in reflexology group (Table 2). No complications were seen in any of reflexology group.

## Discussion

There has been relatively little research in the area of reflexology in pregnancy per se. Randomized controlled trials are the ideal method to explore the effectiveness of clinical interventions. The objective of the present study was to determine the association of pain intensity and labor length with reflexology, as a mild and noninvasive technique of pain management. Our results indicate that the intensity of labor pain was significantly lower in reflexology group in all stages of the study compared with support and routine care groups.

Bering Liisberg in a study on the effect of reflexology on the labor outcome found that out of 68 pregnant women who chose reflexology, 61 (89.71%) expressed that reflexology was helpful in reducing the pain, 6 (8.82%) did not feel any effect and one sub-ject felt increased pain in response to reflexology [17].

The results of a study to investigate whether foot reflexology attenuates acute pain in human volunteers showed that reflexology increased both pain threshold and tolerance in human volunteers exposed to acute pain. These findings indicated the possibility of using reflexology in the management of pain[18]. However, in a retrospective cohort study on antenatal reflexology, the only statistically significant difference between groups was the lack of Entonox usage in the reflexology group; 27 (54%) of the reflexology group used Entonox in comparison to 73 (73%) in the lowrisk comparison group, although the use of opiates and epidurals was the same as the control group. The difference between groups was not significant for the mean duration of labor. The mean duration of labor was very similar between the two groups. The lowrisk comparison group had a mean of 08:27 hours and the reflexology group 08:46 hours demonstrating no significant difference. The mean duration of labour within each group was found to be shorter for those women who had four or more reflexology treatments[19].

The differences in the results of this study and index study might be due to differences in the methodology used and number of cases studied. Motha and McGrath[20] in a similar research observed that the ef-fects of reflexology on labor outcomes were perceived as outstanding and both physical symptoms experienced by pregnant mothers as well as length of labor were reduced. The 20-25 years old subjects had an average duration of first stage labor of 5 or 6 hours. The 26-30 years old patients seemed to have the longest labors. In total, the average duration of first stage was 5 hours, second stage 16 minutes, and third stage 7 minutes. This is compared to textbook figures of 16 to 24 hours of first stage, and, 1 to 2 hours of second stage.

This study demonstrated that reflexology could decrease the duration of first, second and third stages of labor as well as alleviate intensity of labor pain. Therefore, the use of reflexology in maternity care appears to demonstrate high levels of maternal and even staff satisfaction. Reflexology is a relatively new area of care in midwifery and can help to reduce the cumulative rate of elective caesarean section which is mainly due to fear of vaginal delivery. It is a simple and convenient technique that requires no hardware tools, just with the aid of adequately trained, with the aim of reducing the labor pain of pregnant mothers.
